# The Process-Interaction-Model: a common representation of rule-based and logical models allows studying signal transduction on different levels of detail

**DOI:** 10.1186/1471-2105-13-251

**Published:** 2012-09-28

**Authors:** Katrin Kolczyk, Regina Samaga, Holger Conzelmann, Sebastian Mirschel, Carsten Conradi

**Affiliations:** 1 Max Planck Institute Magdeburg, 39106 Magdeburg, Sandtorstr. 1, Germany

## Abstract

**Background:**

Signaling systems typically involve large, structured molecules each consisting of a large number of subunits called molecule domains. In modeling such systems these domains can be considered as the main players. In order to handle the resulting combinatorial complexity, rule-based modeling has been established as the tool of choice. In contrast to the detailed quantitative rule-based modeling, qualitative modeling approaches like logical modeling rely solely on the network structure and are particularly useful for analyzing structural and functional properties of signaling systems.

**Results:**

We introduce the Process-Interaction-Model (PIM) concept. It defines a common representation (or basis) of rule-based models and site-specific logical models, and, furthermore, includes methods to derive models of both types from a given PIM. A PIM is based on directed graphs with nodes representing processes like post-translational modifications or binding processes and edges representing the interactions among processes. The applicability of the concept has been demonstrated by applying it to a model describing EGF insulin crosstalk. A prototypic implementation of the PIM concept has been integrated in the modeling software ProMoT.

**Conclusions:**

The PIM concept provides a common basis for two modeling formalisms tailored to the study of signaling systems: a quantitative (rule-based) and a qualitative (logical) modeling formalism. Every PIM is a compact specification of a rule-based model and facilitates the systematic set-up of a rule-based model, while at the same time facilitating the automatic generation of a site-specific logical model. Consequently, modifications can be made on the underlying basis and then be propagated into the different model specifications – ensuring consistency of all models, regardless of the modeling formalism. This facilitates the analysis of a system on different levels of detail as it guarantees the application of established simulation and analysis methods to consistent descriptions (rule-based and logical) of a particular signaling system.

## Background

Understanding intracellular signaling is one of the major challenges in Systems Biology [1] that is complicated by the nature of signaling molecules themselves: many signaling molecules, in particular receptor molecules, are large structured proteins consisting of several interacting subunits. These subunits, also called domains, usually contain one site which can form a bond with other proteins and/or be subject to post-translational modifications. Hence, each site can take different states. The state of a molecule is defined by the states of its sites (e.g. a receptor is phosphorylated at a particular site and unphosphorylated at another site). If one is interested in the early events of signaling, then realistic descriptions of signaling systems have to reflect this protein structure, at least in part. Hence, already Pawson and Nash proposed to consider the domains of molecules instead of complete molecules as the main players in signaling networks [2].

In modeling approaches, utilizing this point of view, every possible state of a protein is described by a variable of its own. As signaling systems contain many such molecules, each with a large number of domains, one often faces a combinatorial explosion of the number of states [3]. For example, in a *complete description* (i.e. a description incorporating all possible states of all molecule domains), a model of a protein with *n* phosphorylation sites contains 2^*n *^variables. If each site can also be bound by other molecules, the number of required variables increases to 3^*n*^.

In signaling systems composed (mainly) of such structured proteins, subsets of protein states often share common characteristics: for example, if the binding of receptor and ligand occurs with the same kinetic constants, regardless of the phosphorylation state of a different site. In a complete description this binding reaction has to be specified at least twice, with identical rate constants (once for the phosphorylated and once for the unphosphorylated receptor state). This redundant specification makes model set-up complicated and model analysis difficult and thus increases the probability of a model failing to be internally consistent.

Recently, rule-based modeling has been established as the tool of choice to handle this combinatorial complexity. Given a model in a rule-based formalism, quantitative predictions are in general easy to obtain – either via generation of a quantitative model in the form of ordinary differential equations (which is straightforward) or by direct (stochastic) simulation (see, for example, [4,5]). By using the methods described in [6-8] it is possible to reduce the number of equations in an ODE model derived from a rule-based description without losing any information.

Many biologically relevant questions, however, are not necessarily quantitative but rather qualitative in nature. One might, for example, only be interested in whether or not a ligand can activate a transcription factor at all, or how the activation of a certain species is prevented by a small number of knockouts. More details and further examples can be found in [9-11]. Even though these questions can in principle be answered with the help of quantitative models, qualitative models such as logical models have become the tool of choice for studying these questions as they often require less detailed knowledge. For the set-up and analysis of such models a variety of methods exists that are especially suited for studying causal relationships among species in signaling networks. This kind of analysis is often called ‘Structural and Functional Analysis’ [12-15].

Building a logical model describing all possible states of the structured molecules central to signaling systems faces the same challenges as building an ODE model considering such states. Even though it is in principle possible to build such a model (in a way similar to quantitative models), this is a challenging and error-prone task that is not immune to the combinatorial explosion of the number of states. Hence we propose what we call a *site-specific logical model* that enables a systematic description of processes on sites of molecules similar to the rule-based modeling formalism. Site-specific logical models enable – to the best of our knowledge – for the first time the above-mentioned structural and functional analysis of complete descriptions of signaling systems.

In this contribution we will exemplify that the Process-Interaction-Model (PIM) concept combines the advantages of rule-based modeling and site-specific logical modeling in a common representation. Every PIM incorporates all information that is necessary to build consistent models in the different formalisms. Furthermore, this article will describe a concept that comprises algorithms to generate rule-based and logical models from a PIM. Every PIM can be seen as a compact specification of a rule-based model and facilitates the systematic set-up of a rule-based model, while at the same time facilitating the automatic generation of a site-specific logical model.

In the following two subsections we briefly introduce the main concepts of rule-based and logical modeling required for the PIM concept. The remainder of this article consists of the sections “Results” and “Methods”. In the section “Results”, the basic ideas of the PIM concept are introduced, followed by a brief description of its realization within the ProMoT framework [16] and an application to the early events of EGF and insulin signaling. Details of the underlying algorithms and the potential extension of the PIM concept are discussed in the section “Methods”.

### Rule-based modeling facilitates handling of combinatorial complexity

Rule-based modeling has been established as an efficient way to handle the combinatorial complexity that is characteristic for realistic networks in signal transduction [3]. It is an approach tailored to the set-up of such networks and can be seen as a compact model specification [4]. In rule-based modeling classes of biochemical reactions having the same kinetic parameters are described by *reaction rules* that can be expanded to ordinary differential equations (ODEs) in a straightforward way [4,17,18].

By omitting unnecessary information about not involved molecule domains (“don’t care, don’t write principle”) and by using *patterns*, combinatorial complexity can be handled in a systematic manner. Patterns comprise sets of molecules or molecule complexes sharing common characteristics and describe their states. Such a pattern, for example, can comprise all receptor molecules which have a ligand bound, regardless of the states of other phosphorylation and binding sites (i.e. this pattern describes all receptor-ligand complexes with different phosphorylation and binding states).

Patterns are connected by reaction rules describing the evolution of a system. Each rule contains patterns on the right and left side of a reaction arrow followed by kinetic parameters. Every reaction rule is either reversible or irreversible and describes the change of the state of one or two sites (e.g. in modification processes one site changes from unmodified to modified or in binding processes two sites change from unbound to bound). The affected sites in a rule, that is, the sites which change their state, are called the *reaction center*, while sites that remain unchanged are called the *reaction context*[17]. Rules describe biological facts like “the phosphorylation of the insulin receptor at a particular tyrosine residue occurs at a higher rate if insulin is bound to the receptor.”

Many tools facilitate rule-based modeling, for example, BioNetGen [17], ALC [19] and Kappa [20]. These tools require a text-based specification of rule-based models. BioNetGen additionally uses a graph structure to represent these models [21], where the molecules are represented as building blocks composed of reactive sites and the reaction rules are denoted as graph-rewriting rules. The BioNetGen language (BNGL) is emerging as a quasi-standard for rule-based modeling and several rule-based models have already been published in BNGL [22-25]. Furthermore, BioNetGen offers different simulation opportunities of rule-based models and various interfaces to simulation tools [26-28]. Recently, visualization and annotation guidelines for rule-based models have been proposed [24].

**Figure 1:**
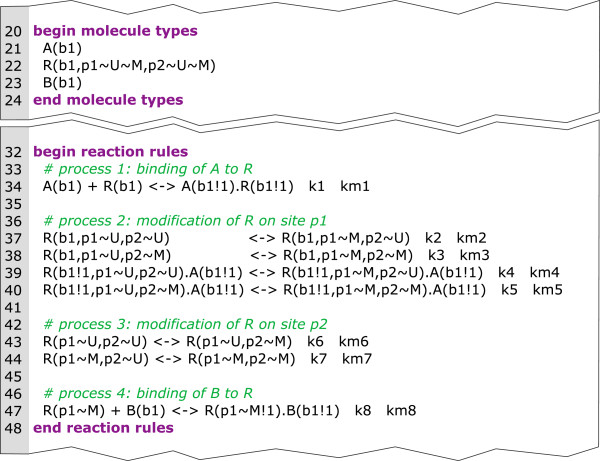
**Source code describing a part of a small example in BNGL.** In BNGL, model elements are specified in blocks enclosed in tags [17]. In this example, the upper block (lines 20–24) specifies the molecules and molecule domains. Three molecules are incorporated: molecule A with one binding site named b1, molecule R with the binding site b1 and the two modification sites p1 and p2 and molecule B with binding site b1. The lower block (lines 32–48) contains the reaction rules with patterns on the left and right of the <-> -symbols and kinetic parameters at the end of each reaction rule. For a complete specification of BNGL see [17].

Figure 1 depicts a part of a rule-based model for a small example system in BNGL. Eight rules (lines 32–48) describe the evolution of the small system. The first rule specifies the binding of molecule A to molecule R. Rules 2 to 5 specify the modification of molecule R on site p1. Each of the four rules describes the modification reaction in a different reaction context. Rule 6 and 7 specify the modification of molecule R on site p2 and rule 8 describes the binding of molecule B to the molecule R.

### Logical modeling facilitates understanding of causal relationships

Qualitative modeling approaches have been emerging as relevant complements to dynamic modeling as they require less detailed knowledge about kinetic laws and parameters while at the same time allowing the study of important structural and functional properties of the system. An example are logical models. Originally used to describe random networks [29] or gene regulatory networks of moderate size [30-33], logical modeling has been established as a valuable tool for the analysis of signaling pathways [9-11,34,35].

For the set-up and analysis of the site-specific logical models presented herein the logical modeling framework introduced in [12] is used. This formalism is tailored to the study of qualitative input-output responses of signaling networks. Biological species such as ligands, receptors, adaptor proteins, or kinases are represented as nodes of the logical network. Each of these nodes has an associated logical state indicating whether the species is active/present (1) or not (0). As the state of a node can also be undefined/unknown (*) a three-valued logic is used. Logical operations on the network nodes represent the signaling events and are given in disjunctive normal form. Besides the logical operators AND, OR and NOT, operators with incomplete truth table (ITT gates) can be utilized in those cases where no decision whether an AND or OR gate should be used can be made [10]. The logical model is represented as a logical interaction hypergraph [12] and methods for the analysis of these networks are implemented in the software *CellNetAnalyzer*[13]. The main difference to the site-specific logical model proposed herein is that states in the latter represent the states of molecule domains instead of molecules themselves.

## Results

In this section we demonstrate that PIM construction is straightforward given graphical representations commonly used in Systems Biology. It is organized as follows: in section “PIM definition and construction” the formal definitions are given. In the sections “A PIM facilitates rule-based model building” and “A PIM uncovers the logic of rule-based models” it is explained how both model types (rule-based and logical) can be derived from a PIM. In the section “Implementation of the PIM concept” we briefly discuss how the concept is realized in the software ProMoT[16] and the section “Application to insulin and EGF signaling” finally demonstrates the applicability and the benefits of a PIM by applying it to the model presented in [7].

### PIM definition and construction

A PIM can be defined for every signaling system consisting of reactions described by mass action kinetics. Obviously, many existing models contain non-mass action kinetics (e.g. convenience kinetics characterizing regulatory feedbacks). These are not directly amenable by the PIM concept. However, we are convinced that this is not a severe limitation, as by modeling such reactions in greater detail it is often possible to replace a reaction with non-mass action kinetics by a network of reactions on the mass action level (many examples can be found in [36]). Moreover, PIMs are expected to be used in modeling early events in signaling pathways; such systems are often modeled in great enough detail to justify mass action kinetics.

A PIM is represented by a directed graph with nodes representing processes like post-translational modification, binding and so on. Edges represent interactions among processes. An edge is added between two processes if a process occurs with different kinetic parameters depending on the occurrence of the other process (e.g. a modification process on a particular site of a receptor is described by different reaction rates, depending on whether or not a ligand is bound). An interaction is either *unidirectional*, *bidirectional* or *all-or-none*. The latter type of interaction can be used to describe a situation where a process can occur only after another process has occurred (e.g. the binding at a phosphorylation site can only occur after the site has been phosphorylated). This type of interaction has been introduced in [6,19] and is employed for model reduction purposes.

**Figure 2:**
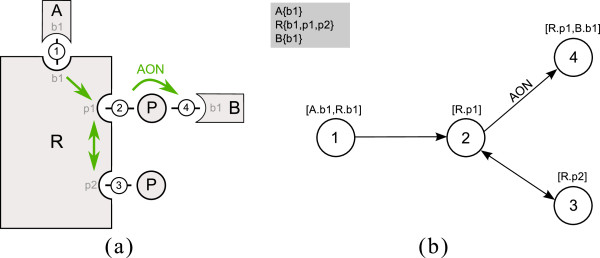
**The Process-Interaction-Model for a small example.** In **(a)** a schematic diagram is shown, and in **(b)** the corresponding graph of the PIM for a small example system is given. Four processes are considered; 1: binding of molecule A (with site b1) to molecule R (site b1), 2: modification (e.g. phosphorylation) of site p1 of molecule R, 3: modification (e.g. phosphorylation) of site p2 of molecule R, 4: binding of molecule B with site b1 to phosphorylated R. Influences among the processes are depicted by green arrows in (a) and black arrows in (b). Binding process 1 has an influence on modification process 2. Process 2 and 3 mutually influence each other. Binding process 4 can only occur if the molecule R has been phosphorylated previously. This is emphasized by the arrow label AON (all-or-none). In the gray box at the upper left side next to the graph the molecules and their sites involved in the system are listed. Molecule A and B each have one binding site b1, the molecule R comprises one binding site b1 and two modification sites p1 and p2.

For a first presentation of these ideas see Figure 2, where a schematic representation of the small introductory example from Figure 1 and the corresponding PIM are depicted. This system consists of four processes. They are represented by four nodes in the PIM (numbered circles). The molecules and sites affected by the processes are depicted as a comma separated list in parenthesis above the nodes. Molecule and site name are separated by a dot. Binding processes have two molecule and site assignments; modification processes have one assignment.

In the context of combinatorial reaction networks, processes and interactions are already introduced in [6,7,19] and a graph with nodes representing processes and edges representing interactions is used in [7]. While in [6,7,19] the focus is on reduction of models of combinatorial reaction networks, the PIM concept focuses on the set-up of two consistent models in different formalisms.

#### The PIM concept is closely related to rule-based modeling

In rule-based modeling one often faces the situation that several rules with the same reaction center but different reaction context are necessary to describe a process. In a PIM every node represents a reaction center and the incoming edges represent the contextual information. Hence, the PIM concept is closely related to rule-based modeling as every process node can be interpreted as an aggregation of reaction rules with the same reaction center and the contextual information of a process node comprises the reaction context of every rule involving that reaction center. This merits our claim that every PIM is a compact representation of a rule-based model. For example, in Figure 2 process node 1 corresponds to the first reaction rule in Figure 1 (i.e. the binding of molecule A and R), process node 2 corresponds to reaction rules 2 to 5 describing the modification of molecule R at site p1 under different conditions (i.e. depending on whether or not the binding of A and R has previously taken place and whether or not molecule R has been modified at p2). Process node 3 corresponds to the reaction rules 6 and 7 (i.e. the modification of molecule R at site p2). And process node 4 corresponds to reaction rule 8 (i.e. the binding of B at the modified site p1 of molecule R).

#### A process node represents a process in different reaction context

The main processes in signal transduction are *binding processes* and *modification processes*. Every process node has assigned information about involved molecules and sites. Consequently, binding processes have assigned two molecules and sites and modification processes have assigned a single molecule and site. Additional process types are defined and will be described in detail in the section “Methods”.

**Figure 3:**
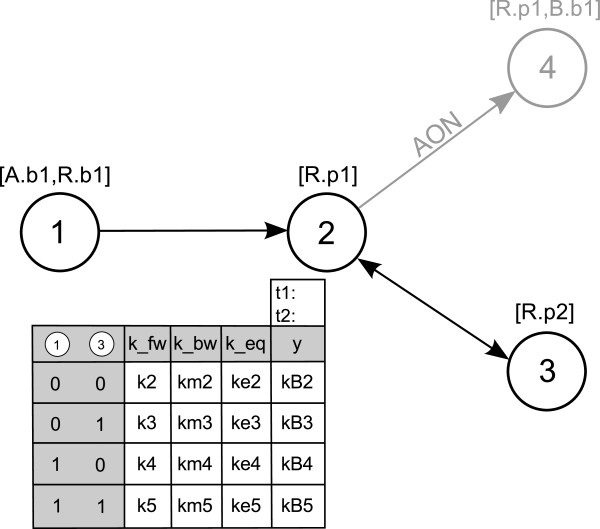
**PIM for the small example depicted in Figure**2**with parameter table for process node 2.** Process node 2 has two incoming edges, one from process node 1 and one from process node 3. There is no incoming edge originating from process node 4, hence, this node is not needed to construct the parameter table and it is consequently shaded in the figure. (For a description of the meaning of the rows and columns see the main text).

Every process can occur in varying reaction context, depending on the status of other processes exerting an influence on it (we call those processes *preceding processes* and note that they are uniquely identifiable as the nodes where the incoming edges of a process node originate). This contextual information defines a parameter table for every process node: each row of the parameter table represents a particular reaction context, that is, a combination of the occurrence of preceding processes (see Figure 3). The number of incoming edges to a process node (*#in* for short) determines the number of columns: the table has *#in*+4 columns, one for each incoming edge, one for the forward rate constant *k*_*fw*_, the backward rate constant *k*_*bw*_, the equilibrium constant $k_{\text{eq}}=\frac{k_{\text{fw}}}{k_{\text{bw}}}$ and the column *y*. Forward and backward rate constants ‘characterize’ the mass action kinetics of the process in a particular context. By default, we assume all processes to be reversible, that is, *k*_*fw *_≠ 0 and *k*_*bw *_≠ 0. Hence, association and dissociation of molecule complexes have to be described as one (reversible) binding process. If a process has to be defined as irreversible, the process can only be irreversible as a whole. In this case, the parameter table does not contain columns for the backward rate constant and equilibrium constant. We do not allow the forward rate constant *k*_*fw*_ to be zero at all. Hence, it is not directly possible to describe dissociation reactions alone (i.e. reactions with one reactant and two products). This limitation is not very strict as from our point of view dissociation without prior binding is not realistic but nonetheless this can be approximated by a reversible binding process with small association and large dissociation constants.

Column *y* stores the information whether the process represented by the node itself has occurred (*y *= 1) or not (*y *= 0). The value of *y* is either determined by the equilibrium constant *k*_*eq*_ (for reversible processes) or the forward rate constant (for irreversible processes), as is described below. The columns representing incoming edges contain logical values denoting the fact that the preceding process has or has not occurred (1 denotes ‘process has occurred’, 0 denotes ‘process has not occurred’). Figure 3 depicts the PIM for the small example in Figure 2 and the parameter table of process node 2. The column labeled 1 indicates the occurrence of process 1 and the column labeled 3 indicates the occurrence of process 3.

#### Parameter tables are related to truth tables

Note that column *y* can be interpreted as an *output* column associated to a process node and indicates if the process is considered as ‘has occurred’ (*y *= 1) or ‘has not occurred’(*y *= 0) for a given combination of input values representing a certain reaction context. Hence, the parameter tables are similar to truth tables, where the inputs for the table are “a previous process has occurred or not”. As in general all combinations have to be accounted for, the table has 2^*#in*^ rows.

To decide about the occurrence of a reaction and thereby the values of the output column, two threshold values *t*1 <* t*2 are introduced. If the process is reversible, the equilibrium constant defined as the quotient of the forward and the backward rate constant of each reaction is used (following from the law of mass action). If the equilibrium constant is greater than or equal to the upper threshold (*k*_*eq *_≥* t*2), we regard the reaction as ‘has occurred’ and set *y *= 1. If the equilibrium constant is equal to or less than the lower threshold (*k*_*eq *_≤* t*1), we regard the reaction as ‘has not occurred’ and set *y *= 0. If *t*1 <* k*_*eq *_<* t*2, neither is the case (*y *= ∗/*unknown*). If the process is defined as irreversible, we use the forward rate constant to decide about the output of the reaction. If *k*_*fw *_≤* t*1, we regard the reaction as ‘ has not occurred’ and set *y *= 0, if *k*_*fw *_≥* t*2, we regard the reaction as ‘ has not occurred’ and set *y *= 1 and if *k*_*fw*_ lies between the two defined thresholds, the output is unknown (*y *= ∗/*unknown*).

This assignment of output values is based on the following idea: we compare the equilibrium constants of the same reaction under different conditions (i.e. in different context) and interpret the relative size of the equilibrium constant as a measure of the influence the reaction context exerts on the outcome of the process. The thresholds are thus a means to reflect this influence of the reaction context and can be chosen for each process individually. Moreover, threshold values will determine topology and logical function(s) of the site-specific logical model.

The choice of thresholds will in general be based on the biological intuition of each modeler as it reflects a judgment about the influence of the reaction context on the process outcome. Hence, threshold choice is one of the most delicate steps in setting up a PIM and one that can, by its nature, not be cast in rigorous rules. In general, it is advisable to study the effect of different threshold choices on the results of a subsequent structural analysis of the site-specific logical model (as we have done in section “Application to insulin and EGF signaling”). It can sometimes be advisable to start with identical thresholds for all or certain subgroups of the processes and refine those later on, based on the structural analysis.

#### How to set up a PIM

**Figure 4:**
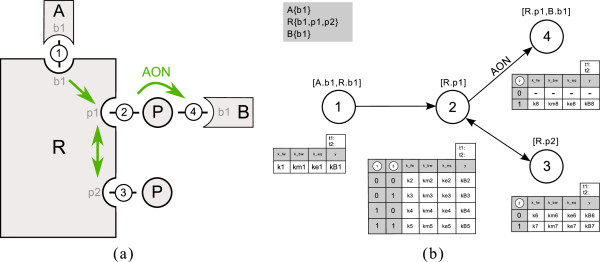
**Complete PIM for the small example.** In **(a)** the cartoon for the small example system is depicted. In **(b)** the corresponding PIM including all information is shown. The molecules and their sites are listed in the gray box. Numbered circles represent the process nodes. Appropriate molecules and sites are indicated in square brackets above the nodes. Nodes are connected by directed edges representing the interactions and each node has an assigned parameter table.

In general, a PIM is set up in a four-step process: In the first step, the molecules and sites which occur in the system have to be defined. The second step is the definition of process nodes. Depending on the type (e.g. binding or modification), involved molecules and sites have to be assigned to the nodes. In the third step process nodes are connected by directed edges representing the interactions. The number of incoming edges to the nodes defines the structure of the parameter tables which have to be filled up with parameters as the final step. Figure 4 shows the complete PIM for our small example system. A prototypic implementation in ProMoT facilitates the four set-up steps (see section “Implementation of the PIM concept”).

### A PIM facilitates rule-based model building

As described in section “The PIM concept is closely related to rule-based modeling”, the PIM concept is strongly related to rule-based modeling. In the generation of a rule-based model, the information about the reaction center can be extracted from a process node; the reaction context in a particular rule is determined by a combination of the occurrence of preceding processes. Kinetic parameters for the combination are taken from the parameter table of the process node. In a PIM, forward and backward rate constants are defined to characterize mass action kinetics of the process. These parameters can be transferred to corresponding reversible reaction rules. If the process is considered to be irreversible, the forward rate constant is added behind the corresponding irreversible reaction rule obtained from the PIM. Furthermore, no units can be defined explicitly in a PIM but parameters are assumed to be specified in consistent units and should be expressed on a per molecule per cell basis.

For a complete rule-based model, the specification of initially existing species and their concentrations is required. For the rule-based models obtained from a PIM, basic (i.e. not complexed) molecules with all sites in unmodified state are assumed. The concentrations are initially set to the value ‘1’ but should be altered afterwards.

The systematic specification of information about involved molecules and their affected sites in process nodes opens up new possibilities in investigating quantitative models. Processes involving particular proteins can easily be omitted in the generation of rule-based models. This greatly simplifies the study of scenarios involving only subsets of proteins (e.g. if a molecule is missing in a model, rules involving this molecule don’t have to be generated). Of course, the study of such scenarios is also possible on the level of reaction rules. But this requires testing every rule, whether or not it involves certain proteins. In a PIM one only has to test each node.

**Figure 5:**
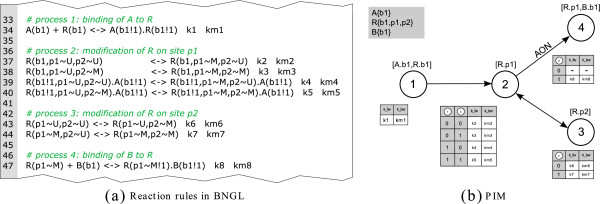
**Generation of reaction rules from a PIM.** In **(a)** the reaction rule block of the BNGL model generated from the PIM in **(b)** is depicted. In the PIM shown in (b) node 1 has no incoming edges, as it does not depend on the occurrence of any other process. Hence one reaction rule is generated. Process 2 depends on process 1 and 3 and all combinations have to be considered. Hence, four reaction rules are derived with different reaction context. Process 3 depends only on process 2. Hence, two reaction rules are generated. Process 4 has an incoming all-or-none interaction from process 2, therefore only the case that process 2 has occurred previously has to be considered and the outcome is one reaction rule.

As BioNetGen is emerging as a quasi-standard for rule-based modeling and as BioNetGen provides all required functionality, we use BNGL as format for rule-based models. The reaction rules for the small example in Figure 4 are given in Figure 5.

### A PIM uncovers the logic of rule-based models

As mentioned in the section “Background”, logical models consist of nodes, each equipped with a logical function. A convenient way to derive a logical model is therefore to begin with an interaction graph, followed by the assignment of a logical function to each of its nodes (called *L-nodes* to avoid confusion with the *P-nodes* of the PIM).

There are many ways to derive a logical model from a PIM. Arguably the easiest way is to create an L-node for every process and use the parameter table belonging to each P-node as truth table defining the logical function of that L-node. This, however, is not what is proposed here because such an interaction graph would not contain information about the connection of molecule domains (i.e. the information that two or more processes occur at the same molecule). Instead, we propose the site-specific logical model mentioned above. This model incorporates information about molecule structure and hence allows capturing more of the biological intuition usually present in a cartoon than is possible with a PIM alone. In particular, a site-specific logical model allows uncovering and visualizing the structure of molecules and their interactions. The construction of this site-specific logical model is described as a two-step process below: first an interaction graph is derived and in a subsequent step each node of the interaction graph is equipped with a logical function.

#### The interaction graph of a site-specific logical model

In order to build the interaction graph, an L-node is created for every site of every molecule and an additional L-node is created for each molecule representing its basal activity. This basal activity connects all L-nodes representing sites of the same molecule and is used to encode the presence/absence of a molecule in different (simulation) scenarios. Later on, in performing logical analysis, L-nodes representing basal activity will serve as inputs.

**Figure 6:**
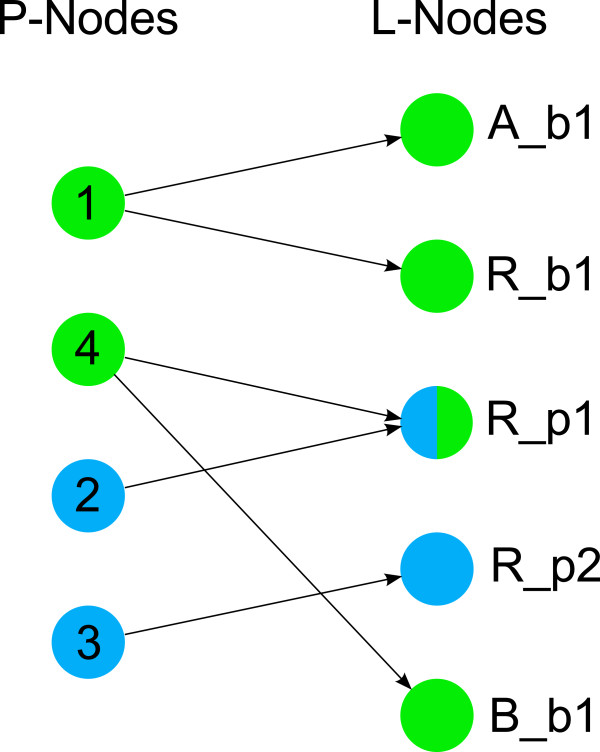
**L-nodes arising from P-nodes of the PIM depicted in Figure**4**.** Binding process 1 and 4 give rise to the L-node pairs (A_b1, R_b1) and (R_p1, B_b1); modification process 2 and 3 give rise to the unique L-nodes R_p1 and R_p2. One can clearly distinguish three types of L-nodes: those arising from a P-node corresponding to a binding process (green), those arising from a P-node corresponding to a modification process (blue) and those arising from both a binding and a modification process (blue and green). The construction of the interaction graph is based on a one-to-one correspondence between P-nodes and a subset of the L-nodes (see main text). To this end, observe that as the L-node R_p1 arises from two processes (binding process 4 and modification process 2), the L-node pair (R_p1, B_b1) can be brought into a one-to-one connection with the P-nodes 2 and 4, while the L-node pair (A_b1, R_b1) cannot be brought into a one-to-one connection with two P-nodes.

In general, the interaction graph will contain more L-nodes than the PIM (it is derived from) contains P-nodes. However, there is an intimate connection between L-nodes and P-nodes (see Figure 6): 

• Every P-node representing a modification process gives rise to a **unique** L-node representing a modification site (as modification processes involve only a unique site).

• Every P-node representing a binding process gives rise to **a pair of** L-nodes representing the binding sites of the involved molecules (as binding processes always involve two binding sites, one from each molecule).

To prepare for the creation of edges, we introduce the *corresponds-to relation* that links L-nodes with P-nodes and L-nodes with L-nodes in a unique way. It is defined for all L-nodes arising from P-nodes as described in Table 1.

**Table 1:** Definition of the corresponds-to relation

**Process(es)**	**P-node(s)**	**L-node(s)**	**Corresponds-to relation**
Modification	*p*_*i*_ …modification process	*L*_*i*_	*L*_*i*_ corresponds to *p*_*i*_
Binding with prior modification	*p*_*i*_ …binding process,	$L_{i}^{\left(1\right)}$ …modification and binding site of molecule one,	$L_{i}^{\left(1\right)}$ corresponds to *p*_*j*_,
	*p*_*j*_ …modification process	$L_{i}^{\left(2\right)}$ …binding site of molecule two	$L_{i}^{\left(2\right)}$ corresponds to *p*_*i*_
Binding without prior modification	*p*_*i*_ …binding process	$L_{i}^{\left(1\right)}$ …binding site of molecule one,	$L_{i}^{\left(1\right)}$ corresponds to *p*_*i*_,
		$L_{i}^{\left(2\right)}$ …binding site of molecule two	$L_{i}^{\left(2\right)}$ corresponds to $L_{i}^{\left(1\right)}$

The corresponds-to relation establishes a one-to-one relation between a subset of the L-nodes and the P-nodes and between two subsets of the L-nodes (for an example see Figure 6).

**Figure 7:**
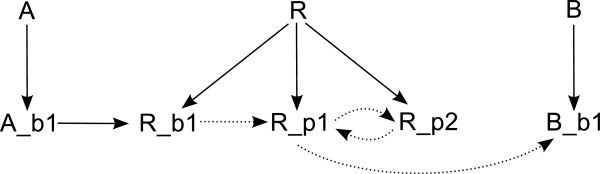
**Interaction graph derived from the PIM depicted in Figure**4**.** The L-nodes A, R and B represent the basal activity of the molecules; the remaining L-nodes represent binding and modification sites. Solid edges denote activations; dotted edges can have a positive or negative sign. L-node A_b1 and R_b1 both arise from the binding process represented by P-node 1 in Figure 4 and neither A_b1 nor R_b1 is subject to a modification process. Hence, one is free to associate either A_b1 or R_b1 to P-node 1 (here L-node R_b1 is chosen and consequently an activating (solid) edge is inserted from A_b1 to R_b1, see main text). L-node R_b1 corresponds to P-node 1 in Figure 4, L-node R_p1 to P-node 2, L-node R_p2 to P-node 3 and L-node B_b1 to P-node 4; the dotted edges correspond to edges between these P-nodes in Figure 4.

In the interaction graph two different types of edges are used: *activating edges* (displayed using solid lines, see Figure 7) and *unsigned edges* that can either be inhibiting or activating, depending on the logical function associated to the L-node at the head of the edge (displayed using dotted lines, see Figure 7).

*Unsigned edges* are in a one-to-one correspondence to edges between P-nodes in the PIM. Their introduction is based on the corresponds-to relation established in Table 1. In the interaction graph an unsigned edge is created between two L-nodes *L*_*i*_ and *L*_*j*_ if the following conditions are satisfied: 

1. *L*_*i*_ and *L*_*j*_ correspond to two P-nodes *p*_*i*_ and *p*_*j*_ (see Table 1).

2. There exists an edge between *p*_*i*_ and *p*_*j*_.

In this case the edge between *L*_*i*_ and *L*_*j*_ has the orientation of the edge between *p*_*i*_ and *p*_*j*_: from *L*_*i*_ to *L*_*j*_ if the edge in the PIM is from *p*_*i*_ to *p*_*j*_ and from *L*_*j*_ to *L*_*i*_ if the edge in the PIM is from *p*_*j*_ to *p*_*i*_ (the dotted edges in Figure 7). *Activating edges* are created between two L-nodes *L*_*i*_ and *L*_*j*_ in one of the following two cases: 

1. One of the two nodes, say *L*_*i*_, represents the basal activity of a molecule and the other node *L*_*j*_ represents a site of this molecule. In this case an edge is created from *L*_*i*_ to *L*_*j*_. These activating edges represent the molecule structure. In a subsequent logical analysis this allows, for example, removal of a molecule (and all of its sites) by assigning a value ‘0’ to the L-node representing the basal activity.

2. Both L-nodes are connected by the corresponds-to relation. Assume *L*_*i*_ corresponds to *L*_*j*_ (see Table 1, row 3). Then an edge is created from *L*_*i*_ to *L*_*j*_. This situation can only occur if both L-nodes arise from a binding process without prior modification (e.g. the activating edge from A_b1 to R_b1 in Figure 7).

In the latter case the orientation of the activating edge depends on the decision which of the two L-nodes corresponds to the P-node representing the binding process (see the last row in Table 1). This choice is **arbitrary** and **does not affect** the results of the subsequent logical analysis, because, by definition, *L*_*i*_ has exactly one incoming activating edge from the L-node representing the basal activity. Hence, in analysis *L*_*i*_ passes the value of the L-node representing the basal activity to *L*_*j*_.

#### From interaction graphs to site-specific logical models: equipping L-nodes with logical functions

**Figure 8:**
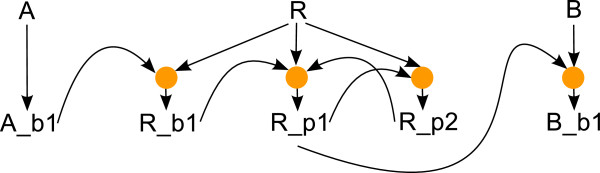
**Logical model for the small example model derived from the PIM depicted in Figure**4**.** Several incoming edges to a node in the interaction graph (Figure 7) are combined by a logical function represented as an orange circle.

To obtain a site-specific logical model from the interaction graph, every L-node has to be equipped with a logical function connecting all incoming edges (see Figure 8). To this end, note that we may distinguish between two types of nodes, depending on the incoming edges: nodes having unsigned (dotted) incoming edges and nodes without unsigned incoming edges. Below logical function construction is first described for nodes having no unsigned incoming edges, followed by a description of logical function construction for nodes having unsigned incoming edges.

*Logical function construction for L-nodes without unsigned incoming edges:* For these L-nodes the logical function is a logical AND connecting all inputs. *Logical function construction for L-nodes with unsigned incoming edges:*

**Figure 9:**
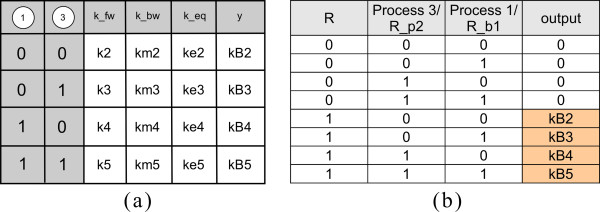
**Transfer of values from parameter tables to truth tables.** The parameter table of P-node 2 (as described in Figure 3) is shown in **(a)**. The truth table for the logical function connecting the incoming edges to L-node R_p1 in Figure 8 is shown in **(b)**.

From the construction of the interaction graph described above it is guaranteed that every L-node of this type is associated to a unique P-node (this is not the case for L-nodes in general, as some of them are associated to a different but unique L-node). The logical function for L-nodes associated to a unique P-node is obtained from the parameter table of that P-node in the following way: as described in section “PIM definition and construction”, such a parameter table can be interpreted as a truth table and the logical function defined by that truth table is the basis for constructing the logical function associated to the L-node. By definition, this logical function has one input variable for every incoming unsigned edge of the L-node (as unsigned incoming edges to L-nodes are in a one-to-one correspondence to incoming edges to P-nodes and as the parameter table has one column for each incoming edge, see section “PIM definition and construction”). Given a hypothetic L-node having only unsigned incoming edges, the logical function defined by the parameter table could be used. In practice, if an L-node has incoming edges, at least one of the edges is an activating one. Each activating incoming edge defines an additional input variable that is joined to the function by a logical AND. Figure 9 shows the parameter table of a P-node in (a) and the truth table of the corresponding L-node in (b).

#### From truth tables to logical functions

The aim is to enable analysis of the site-specific logical model with methods available in *CellNetAnalyzer*[13]. Therefore, the logical functions connecting incoming edges to nodes have to take the form of a sum of products. If 0 and 1 are the only values in the output columns of the respective truth tables, this is equivalent to the disjunctive normal form. Moreover, determination of a logical function from a truth table is straightforward in this case as one may use established algorithms like k-maps (Karnaugh-Veitch [37,38]) or the Quine-McCluskey algorithm [39] to obtain a logical function in disjunctive normal form.

From the previous discussion, however, it is obvious that truth tables containing ‘unknown’-symbols can be associated to an L-node. In this case, the aforementioned algorithms are not applicable (note that the ‘ don’t care’-symbols allowed in k-maps and the Quine-McCluskey algorithm are different from the ‘unknown’-symbols considered here, in turn precluding applicability of these algorithms). Logical functions hence have to be inferred from truth tables involving ‘ unknown’-symbols on a case-by-case basis. To guarantee applicability of the methods proposed in [13], it is recommended to use ITT gates [10] to accommodate for the ‘unknown’-symbols: for example, if the first row (all inputs equal 0) has the output 0 and the last row (all inputs equal 1) has the output 1.

### Implementation of the PIM concept

**Figure 10:**
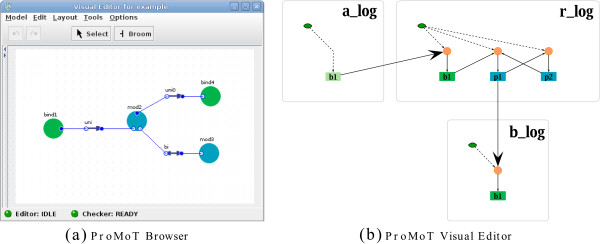
**Implementation of the PIM concept.** In **(a)** the PIM of the small example depicted in Figure 4 is shown in the ProMoT Visual Editor. Green circles denote binding processes; blue circles denote modification processes. In **(b)** the modular logical model derived from the PIM in (a) is shown. The ProMoT Visual Explorer facilitates sophisticated graphical representations of logical models. Green and blue filled rounded rectangles represent the processes occurring on the sites of molecules; light green rounded rectangles represent binding sites which do not correspond to a process. Orange circles depict the logical functions connecting the incoming edges to the nodes. As described above, these logical functions are determined by the parameter tables. The small green nodes (with hexagonal shape) connected to the logical functions depict the basal activity of molecules. The module borders are represented by gray rounded rectangles around the nodes belonging to one molecule.

The PIM concept introduced in the previous sections has been realized in the modeling software ProMoT[16,40]. ProMoT is an open-source software and can be downloaded from http://www.mpi-magdeburg.mpg.de/projects/promot. ProMoT is a tool for model set-up and visualization of different model types in application areas like chemical engineering, systems biology and synthetic biology. Different modeling approaches are supported by specialized libraries containing modeling entities. For PIMs a specialized library in combination with suitable graphical representations for the modeling entities has been developed. There the main process types binding and modification are supported. Figure 10 shows two screenshots of the realization of the PIM concept in the modeling software ProMoT.

Furthermore, export functionality has been added to obtain rule-based models in BNGL from PIMs set up in ProMoT. The conversion into logical models is directly done in ProMoT and will be described in more detail in the next section. The software extension supporting PIMs is available upon request and will be contained in a future release.

#### A modular logical model obtained from a PIM enables an intuitive analysis and visualization

One of ProMoT’s key features is the opportunity to set up modular models. Modules are used to structure a model and easily exchange and reuse model parts in the modeling workflow. This feature is facilitated in the generation of logical models from PIMs. One module encapsulates all nodes representing the parts of the same molecule. Hence, every molecule is represented by a module and the interactions with other molecules are depicted by arrows across module borders. Figure 10(b) shows the modular logical model for the small example depicted in Figure 8 in the ProMoT Visual Explorer. ProMoT comprises functionality which enables to obtain logical models intended for the analysis in *CellNetAnalyzer* combined with suitable graphical representations.

### Application to insulin and EGF signaling

**Figure 11:**
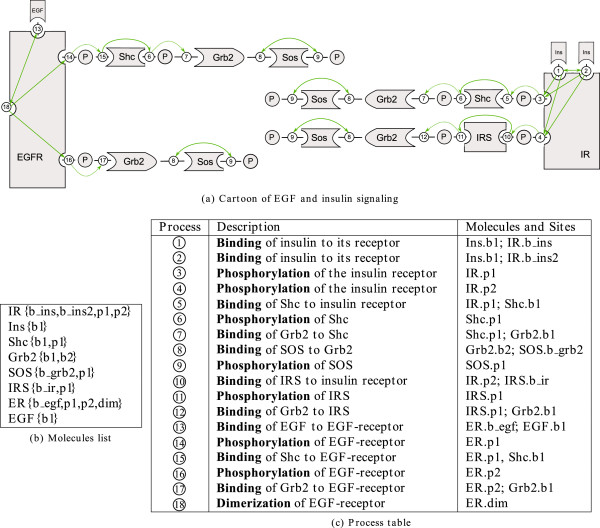
**Early events in insulin and EGF signaling as considered in **[7]**.** The cartoon shown in **(a)** has been adopted from [7] and processes (bulleted numbers) and interactions (green arrows) needed for PIM construction have been included. **(b)** List of molecules extracted from the cartoon in (a). The sites of each protein are listed in curly braces. **(c)** Processes considered in (a) and their description: The first column enumerates the processes, the second column gives a short textual description and the third column lists molecules and sites involved in the process (one molecule and site for modification and polymerization processes, two for binding processes).

To demonstrate the applicability of our concept, a PIM describing early events in EGF and insulin signaling has been constructed. This PIM is based on the cartoon depicted in Figure 11(a) that has been adopted from [7]. In Figure 11(a) the EGF receptor is shown with four domains, one extracellular domain containing the ligand binding site, one domain containing a dimerization site, and two intracellular domains containing binding sites for the effector proteins Shc and Grb2. The insulin receptor is also considered with four domains, two with binding sites for insulin molecules, and two containing binding sites for the adaptor Shc and IRS. As in [7], further domains are neglected to avoid combinatorial explosion of feasible receptor species.

**Figure 12:**
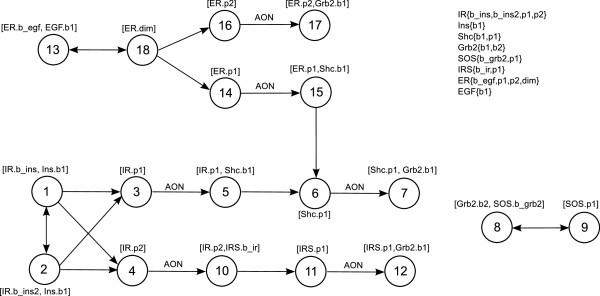
**PIM for EGF insulin crosstalk.** The shown PIM describes the early events in insulin and EGF signaling and comprises 18 process nodes connected by different interaction types.

From Figure 11(a) the molecules, their structure and a total of 18 processes has been identified (see tables in Figure 11(b) and 11(c). Interactions among processes are indicated as green arrows in Figure 11(a). The 18 processes listed in Figure 11(c) are depicted as numbered circles in Figure 12. Node 1 and 2 represent, for example, the binding processes of insulin molecules to their receptor; node 3 and 4 stand for the phosphorylation processes at the two insulin receptor sites for effector binding. The two insulin binding domains influence each other (resulting in the bidirectional edge between node 1 and 2) as well as the other two domains of the receptor (resulting in the edges from node 1 and 2 to 3 and 4). The columns for forward and backward rate constants of the parameter tables have been filled with the help of the reaction rules formulated in ALC language in [7]. The entire PIM including parameter tables can be seen in Additional file 1. From this PIM a rule-based model can be derived in BNGL in a straightforward way. The resulting BNGL file contains 36 reaction rules and is available in Additional file 2.

#### Threshold choice and its effect on the logical model

A site-specific logical model can be derived from the PIM as described above. In doing so, a crucial point is the choice of the thresholds *t*1, *t*2 to discretize the equilibrium parameter. In our particular example, we decided to use the same thresholds for all reactions to limit the number of degrees of freedom. We have chosen *t*1 = 0.01 and *t*2 = 0.1 (Additional file 3 contains the model, readily prepared for the analysis in *CellNetAnalyzer*). To examine the effect of the threshold values on the logical model, we also considered two other model variants where we moved both thresholds to the next larger/smaller value appearing as equilibrium constant (model *M*_*down*_: *t*1 = 0.001, *t*2 = 0.01; model *M*_*up*_: *t*1 = 0.1, *t*2 = 0.25).

**Figure 13:**
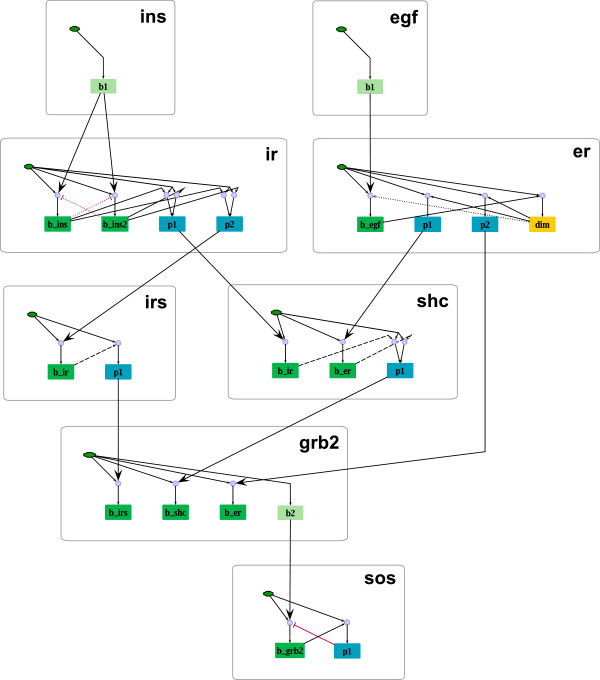
**Site-specific logical model derived from the PIM in Figure**12**.** Nodes representing the basal activity of a molecule are depicted as green hexagons. Blue rounded rectangles stand for nodes corresponding to phosphorylation sites. Dimerization sites are depicted in yellow. Nodes representing binding sites of the respective molecule are depicted as green (if the L-node corresponds to a unique P-node, that is, to a binding process in the PIM) and light green filled rounded rectangles (if the L-node corresponds to a unique L-node; see section “A PIM makes the logic of rule-based models transparent”). Three model variants are illustrated: Model *M*_*down*_ contains all solid arrows, model *M* contains all solid and dashed arrows and model *M*_*up*_ contains all arrows (solid, dashed and dotted). For all three model variants blue circles symbolize AND gates and all incoming edges to a node are by default connected by OR. Black arrows indicate activating, red blunt-ended lines inhibiting influences. As described above, the logical functions have been derived from the parameter tables (see Additional file 1). The module borders are visualized by gray rounded rectangles. The visualization has been created in ProMoT.

The effects are illustrated in Figure 13. One arrives at the following conclusions: 

• If threshold values are increased, the logical model becomes more restrictive, that is, compared to model *M*, model *M*_*up*_ contains additional influences: (1) EGF dimerization becomes necessary for EGF binding in model *M*_*up*_. As EGF binding is in turn necessary for dimerization, neither of the two states can ever be activated in model *M*_*up*_, thus supporting model *M*. (2) The two insulin binding sites on the insulin receptor mutually inhibit each other, that is, insulin can only bind to either site in *M*_*up*_. This indeed reflects the biological situation [41]. Hence, even though (1) clearly argues against increasing the thresholds, (2) seems to indicate that it might be necessary to vary the thresholds of individual processes (e.g. those of process 1 and 2), also accounting for possible parameter uncertainties. In this example, we nevertheless decided to use one threshold value for all processes, not least because in this particular case, the outcome of the logical analysis is to a certain degree independent of whether or not we assume that the two binding sites influence each other.

• If threshold values are decreased, biochemically important interactions are missing: in *M*_*down *_IRS and Shc phosphorylation depend only on the basal activity of the respective molecules. Thus model *M*_*down *_does not account for the fact that both phosphorylation events are induced by preceding binding events, again supporting model *M*.

**Figure 14:**
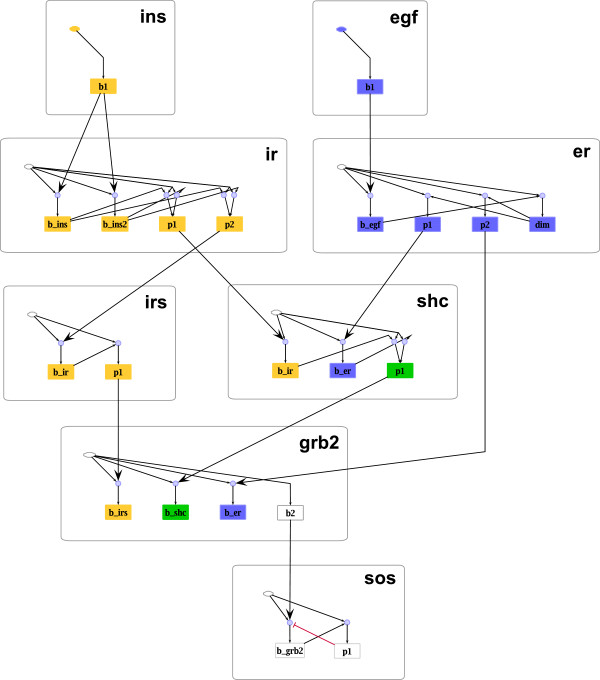
**Species equivalence classes in the site-specific logical EGF insulin model.** The basal activities for insulin and EGF have been undefined, all other basal activities set to the value 1. Thus, for each of the ligand combinations (i.e. EGF and insulin absent, EGF and insulin present, either of them present) all species contained in one equivalence class have the same value in the corresponding logical steady state. Three different equivalence classes can be found for this particular input scenario: Yellow nodes indicate insulin-specific parts of the network (i.e. L-nodes that are on if insulin is present, off if insulin is absent); blue L-nodes indicate EGF-specific parts, and green L-nodes are influenced by insulin as well as EGF. White L-nodes are not contained in any equivalence class. The computation of equivalence classes has been performed in *CellNetAnalyzer*. The results have been re-imported and visualized in ProMoT.

Once a site-specific logical model has been derived from the PIM, structural and functional properties can be analyzed, for example by using the software tool *CellNetAnalyzer*[13]. This is demonstrated by applying two of the available methods to the model *M*: (1) Computation of species equivalence classes [10]. This reveals EGF- and insulin-specific parts of the network together with those parts that are influenced by both pathways (see Figure 14). (2) Computation of minimal intervention sets [12,14], for example to prevent binding of Grb2 to Shc in response to insulin stimulation. The results are given in Table 2 and exemplify that a site-specific logical model can suggest interventions at different levels: by removing whole proteins from the system (Table 2, rows 1–3), as well as by either modifying/removing binding sites (rows 4–6, 8) or by preventing a necessary modification (rows 7, 9).

**Table 2:** Minimal intervention sets to prevent binding of Grb2 to Shc in response to insulin stimulation

	**Minimal intervention set**	**Interpretation**
1	ir.res_ir=0	
2	grb2.res_grb2=0	set basal activity of insulin receptor, Grb2 or Shc to 0, i.e. remove respective species from the system
3	shc.res_shc=0	
4	ins.b1=0	prevent insulin binding to its receptor by blocking the binding site on insulin or by blocking both
5	ir.b_ins=0, ir.b_ins2=0	binding sites on the receptor
6	shc.b_ir=0	prevent Shc binding to insulin receptor by blocking the binding site on Shc or by preventing the
7	ir.p1=0	necessary phosphorylation of the receptor
8	grb2.b_shc=0	prevent Grb2 binding to Shc by blocking the binding site on Grb2 or by preventing the
9	shc.p1=0	necessary phosphorylation of Shc

The nine minimal intervention sets preventing binding of Grb2 to Shc in response to insulin stimulation are given in the rows. The sets are grouped according to their effect on the signaling pathway.

## Conclusions and discussion

We introduced the Process-Interaction-Model (PIM) concept as a means of combining the advantages of rule-based and logical modeling approaches. A PIM is based on a directed graph and incorporates the definition of molecules, domains, processes, interactions, kinetic parameters and logical values. A prototypic implementation of the PIM concept has been integrated in the modeling software ProMoT. At the moment this software-extension is available on demand and will be contained in a future release.

A PIM can be seen as a compact description of rule-based models and the concept thereby facilitates the systematic set-up of such models. Besides rule-based models logical models can be derived from the same basis. Thus the PIM concept enables a systematic and consistent set-up of models in two different specifications. Consequently, the signaling system can be studied on two different levels of detail by applying established simulation and analysis methods to both models. The common basis for the two models has the additional advantage that modifications (e.g. new insights on the structure of signaling systems or changes of parameters) can be made on the basis model and propagated into the model specifications.

When defining a PIM, one faces the same problems as when setting up a rule-based model: one needs to specify rate constants for every reaction and every reaction context. These are often hard to come by. The generation of the site-specific logical model additionally needs values for the thresholds. These can be equally hard to determine. For conventional logical models this information is not necessary, hence, the construction of a site-specific logical model using a PIM can be more involved. A PIM, however, is an efficient means to generate models of two different formalisms in a consistent way. This can more than offset the effort of specifying all parameters.

In the following paragraphs we briefly discuss the potential of the PIM concept.

### A modeling workflow incorporating PIMs

A possible modeling workflow employing the PIM concept starts with the set-up of the PIM. As a second step, a site-specific logical model is generated and the qualitative behavior and structural properties of the signaling system are determined by structural and functional analysis of this model. In step three, a PIM refinement may be required based on the results of step two (i.e. processes have to be changed, interactions have to be added or replaced and/or parameters have to be changed accordingly). Steps two and three have to be repeated until further refinement is unnecessary and the logical model can reproduce experimental data. In step four, a rule-based model is generated and the quantitative behavior of the signaling system is determined by simulation and analysis of the rule-based model or the corresponding ODE model. Further cycles of PIM refinement, generation of the rule-based model and its simulation may be necessary to explain experimental data.

Site-specific logical models obtained with the PIM concept will usually describe signaling events in a very detailed manner. This is justified for early events in signaling systems. A natural extension of the aforementioned steps is therefore an integration of the site-specific logical model into existing logical models describing signaling events further downstream of the receptor.

This modeling workflow is only one possibility to employ the PIM concept for the investigation of signaling systems and will most likely have to be adapted to the problem to be solved.

### PIMs facilitate scenarios for rule-based models

The systematic specification of involved molecules and their affected sites in each process node greatly simplifies the study of scenarios that describe the removal of proteins from the system. This is especially useful for rule-based models where the systematic removal of proteins can be challenging. It is straightforward to generate not only one rule-based model but a family of models. This enables an analysis that has hitherto been restricted to logical models: to study the influence presence/absence of a molecule has on the system.

### PIMs may support model reduction and checking of thermodynamic constraints

In general, quantitative models derived from PIMs result in tremendous ODE systems, thus model reduction is reasonable. The directed graphs used in the PIM concept are similar to the ones used in reduction techniques for rule-based models described in [6,7]. It is in principle possible to adapt and apply these methods to the PIM concept such that both the rule-based specification and the site-specific logical model can be reduced in one step by applying them to the PIM. Furthermore, the systematic assembly of kinetic parameters enables comfortable checking of thermodynamic constraints.

To conclude, the PIM concept offers connections to a variety of established methods. It has the potential to become a valuable tool.

## Methods

In the previous sections we presented the concept of a PIM with the two process types occurring most frequently in signaling systems. We pointed out which information has to be assigned to process nodes representing processes of the type binding and modification: the involved molecules, their affected sites and parameter tables. Furthermore, algorithms for the generation of rule-based and site-specific logical models from a PIM containing these process types have been worked out. In the following, algorithmic details for rule-based and logical model generation will be illustrated for special cases of combinations of these two process types, followed by a discussion on how further process types can be integrated in the PIM concept. Thereby, simple examples will be used to demonstrate how other process types can be represented in the PIM concept and how the algorithms for the generation of rule-based and site-specific logical models can be extended. The complete description of these process types is beyond the scope of the paper.

### Algorithmic details

In the following, special cases of constellations of binding and modification processes will be presented. Peculiarities may arise in the generation of rule-based and site-specific logical model specifications from a PIM.

#### One molecule can bind on different sites which are subject to prior modification

**Figure 15:**
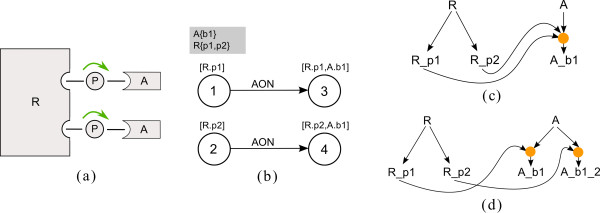
**Special case: One molecule can bind on different sites on another molecule.** In **(a)** the cartoon of the example system is depicted. Molecule A can bind on two different sites on a molecule R; both sites have to be phosphorylated previous to the binding. **(b)** Process node 1 and 2 represent modification processes, process node 3 and 4 the two binding processes. Each modification process on the particular site exerts an influence in form of an all-or-none interaction on the following binding process. In **(c)** one L-node is introduced for each site of a molecule (R_p1, R_p2 and A_b1). Additional L-nodes representing the basal activity of molecule A and R have an activating influence on L-nodes representing the sites. The arrows from R_p1 to A_b1 and from R_p2 to A_b1 represent the two all-or-none interactions among modification and binding processes. In this logical model the two binding processes are represented by a single L-node. As described above, every P-node has to correspond to a unique L-node. Hence, a further L-node has been introduced in the logical model shown in **(d)** to enable a one-to-one correspondence of P-nodes to L-nodes.

In section “Results” we demonstrated that each P-node must have exactly one corresponding L-node. A special case in the derivation of the logical model arises if one molecule can bind to two different sites on another molecule which are both subject to prior modification, that is, two all-or-none interactions point to two binding processes which occur at a common binding site. Thus, the two P-nodes representing these binding processes give rise to the same L-node. Finding a logical function for such an L-node would require information about the common occurrence and interplay of both modifications. This information does not exist. Hence, two P-nodes cannot correspond to the same L-node and a further L-node has to be added. Figure 15 shows an example system with four processes.

#### Mutually exclusive preceding processes

**Figure 16:**
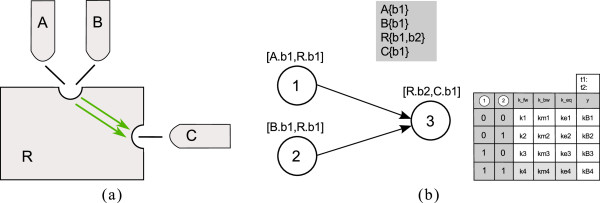
**Special case: Two preceding processes involve a common binding site (and are therewith mutually exclusive).** In **(a)** the cartoon of the example system is depicted; **(b)** depicts the corresponding PIM.

Another special constellation in PIM is the influence two processes involving a common site have onto another process. Figure 16 shows an example. The binding of molecule A and molecule B to the same site on a molecule R influence the binding process of another molecule C to R. In rule-based modeling approaches single molecules are considered. Hence, one molecule can only bind to one other site at a defined time. Figure 16(b) shows the PIM for the system. Node 1 and 2 describe the two binding processes of molecule A and B to R, node 3 describes the binding of C to R which is influenced by the competing binding processes 1 and 2. In a single molecule approach, A can only bind to one site. Hence process 1 and 2 cannot occur simultaneously in rule-based models. This is represented by the grayed fields in the parameter table of process node 3. In the corresponding site-specific logical model process 1 and 2 can occur at the same time. Hence the value in column *y* can be set directly.

### Discussion of additional process types

In intracellular signaling, processes other than binding and modification can occur. Arguably the most important ones are polymerization, synthesis, degradation and change of compartment. Although these process types are currently not implemented, it is in principle possible to incorporate them. Below we briefly discuss how this can be achieved.

#### Polymerization

**Figure 17:**
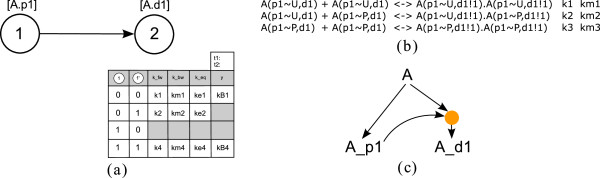
**Example containing a dimerization process. (a)** shows the PIM: Process 1 represents a modification process on molecule A and process 2 a dimerization of two A molecules. The parameter table contains two input columns for the influencing modification process to represent the states of the two monomers. In **(b)** the corresponding reaction rules for process node 2 in BNGL are listed, and **(c)** shows the logical model for the example.

Polymerization of, for example, receptor molecules often plays a role in signaling. We will briefly discuss how dimerization, the simplest type of polymerization, can be integrated in the PIM concept. For heterodimerization, the general *binding* process can be utilized and the processes for each monomer are described with different process nodes. For homodimerization, we need a special concept as the two monomers that dimerize are not necessarily in the same state (e.g. dimerization of ligand-bound and ligand-free receptor or phosphorylated and unphosphorylated monomers). Hence, a new process type for homodimerization has to be introduced. To every node of this type the name of the monomer and the dimerization site have to be associated. For the parameter table the inputs are duplicated to match the states of the two monomers. Figure 17 shows an example containing a dimerization process.

In the parameter table in Figure 17(a) the cases ‘01’ and ‘10’ are identical for homodimerization. Parameters are just needed for one of these cases, thus the gray row means that the fields should not be filled with parameters. Analogous to BioNetGen the association of monomers in the same state (i.e. the first and the last row in the parameter table in Figure 17(a)) is parametrized with 0.5 times the nominal rate constant [21]. Hence, for the logical approach, we have to assume that all representations of the same species are in the same state, either *on* (e.g. phosphorylated in Figure 17) or *off* (e.g. unphosphorylated in Figure 17). Therefore, for the logical model, only the rows for equal monomer sites are considered. The remaining fields in the parameter table in Figure 17(a) are colored in gray.

#### Degradation and synthesis

Degradation is a further process occurring in signaling systems. Here we argue that this process type could be integrated in the PIM concept. Information about the name of the molecule which will be degraded and a parameter table have to be assigned to the process node. As degradation is an irreversible process and BioNetGen has a special concept to describe these reactions [17], kinetic parameters are only needed for the forward direction.

BioNetGen additionally allows specifying the degradation of complexes by adding keywords to the rules [17]. Here we restrict our discussion to the simplest case, the degradation of a single molecule.

**Figure 18:**
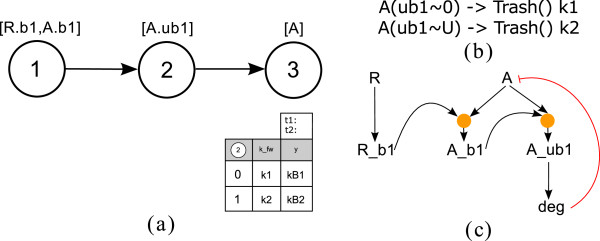
**Example containing a degradation process.** In **(a)** the PIM is shown: Node 1 represents a binding process of molecule A to molecule R; process 2 is a modification (ubiquitination) process on molecule A. Process 3 represents the degradation of molecule A which depends on the previous ubiquitination. The parameter table contains one input column for the influencing modification process. In **(b)** the corresponding reaction rules for process node 3 are listed in BNGL; and **(c)** shows the logical model for the PIM introduced in (a).

In the generation of a site-specific logical model an additional L-node representing the degradation process is inserted. Furthermore, an inhibiting edge from this L-node to the L-node representing the basal activity of the degraded molecule is added. Thus, the L-node for the degradation exerts a negative influence on all subsequent processes. In logical analysis, this inhibiting edge has to be considered with a time delay [12]. In the determination of the logical function connecting the inputs to the degradation process, the thresholds are used to discretize the forward parameter. Figure 18 shows an example containing a degradation process which is influenced by an ubiquitination.

To describe the synthesis of a molecule, another specialized process type has to be included in the PIM concept. A process node of this type has to store information about the newly synthesized molecule, the state of its sites, the additional molecule and if it is synthesized bound or unbound.

Logical models obtained from PIMs which contain synthesis processes do not gain additional information because in the site-specific logical models the basal activity species act as inputs and are set prior to analysis.

#### Change of compartment can be modeled like a modification process

The change of a species localization is a process occurring frequently during the signal transfer in cells; for example, a receptor receiving the signal from extracellular space is internalized (i.e. moves into an endosome). Rule-based models seldom incorporate information about species localization because it raises intricacy of the models. In BioNetGen molecule localization can be treated like a modification. A further site is added and the state of this site represents the localization of a molecule. In complex cases, however, this approach may be error-prone. The modeler has to take care that molecules in complexes change their state of the location together and that molecules can only switch into adjacent compartments. Furthermore, for processes taking place in different compartments, identical reaction rules varying solely in their localization state have to be written down. To overcome this difficulties, BioNetGen has recently incorporated a concept called cBNGL [42]. This approach adds an additional attribute to species and molecules and therewith enables modeling of compartmental organization of cells by storing a directed graph representing the compartment topology. Incorporating this approach into PIMs would require storing additional information. Hence, it is currently not possible to generate rule-based models in cBNGL. Instead, in PIM two different compartment localizations for a molecule can be facilitated by treating compartment changes like modification processes. For that, no special process type is introduced. Some of the disadvantages of modeling compartment changes like modifications inducing error-proneness are overcome by the systematic specification approach followed by PIM. Nevertheless, the modeler has to take care about adjacency of compartments.

## Competing interests

The authors declare that they have no competing interests.

## Author’s contributions

KK, RS, HC designed the PIM concept. KK implemented the concept in the modeling software ProMoT. KK, RS, SM and CC prepared the manuscript jointly. All authors have read and accepted the manuscript.v

## Supplementary Material

Additional file 1 **Process-Interaction-Model of EGF insulin crosstalk (additional information).** Additional file 1 lists the reaction rules and the associated parameters taken from [7] and used to elaborate the EGF insulin crosstalk example in the section “Results”. The process nodes of the PIM are correlated to the reaction rules in a tabular fashion. Furthermore, all parameter tables associated to the process nodes of the PIM are given. Click here for file

Additional file 2 **Input file for the software BioNetGen (EGF insulin crosstalk).** Additional file 2 contains the rule-based model obtained from the PIM describing EGF insulin crosstalk (section Results) and can be used as an input file for BioNetGen. Click here for file

Additional file 3 **Input files for the software*CellNetAnalyzer* (EGF insulin crosstalk).** This archive contains all files of the logical model obtained from the PIM describing EGF insulin crosstalk (section Results) in *CellNetAnalyzer* format. *CellNetAnalyzer* is available from http://www.mpi-magdeburg.mpg.de/projects/cna/cna.html. Click here for file

## References

[B1] Gilbert D Fuss H Gu X Orton R Robinson S Vyshemirsky V Kurth MJ Downes CS Dubitzky W Computational methodologies for modelling, analysis and simulation of signalling networks Brief Bioinform 2006 7 4 339 353 http://dx.doi.org/10.1093/bib/bbl043 10.1093/bib/bbl043 17116646

[B2] Pawson T Nash P Assembly of cell regulatory systems through protein interaction domains Science 2003 300 5618 445 452 http://dx.doi.org/10.1126/science.1083653 10.1126/science.1083653 12702867

[B3] Hlavacek WS Faeder JR Blinov ML Perelson AS Goldstein B The complexity of complexes in signal transduction Biotechnology and Bioengineering 2003 84 7 783 794 http://dx.doi.org/10.1002/bit.10842 10.1002/bit.10842 14708119

[B4] Faeder JR Blinov ML Goldstein B Hlavacek WS Rule-based modeling of biochemical networks Complexity 2005 10 4 22 41 http://dx.doi.org/10.1002/cplx.20074 10.1002/cplx.20074

[B5] Sneddon MW Faeder JR Emonet T Efficient modeling, simulation and coarse-graining of biological complexity with NFsim Nat Methods 2011 8 2 177 183 http://dx.doi.org/10.1038/nmeth.1546 10.1038/nmeth.1546 21186362

[B6] Koschorreck M Conzelmann H Ebert S Ederer M Gilles ED Reduced modeling of signal transduction - a modular approach BMC Bioinformatics 2007 8 336 http://dx.doi.org/10.1186/1471-2105-8-336 10.1186/1471-2105-8-336 17854494 PMC2216040

[B7] Conzelmann H Fey D Gilles ED Exact model reduction of combinatorial reaction networks BMC Syst Biol 2008 2 78 http://dx.doi.org/10.1186/1752-0509-2-78 10.1186/1752-0509-2-78 18755034 PMC2570670

[B8] Borisov NM Chistopolsky AS Faeder JR Kholodenko BN Domain-oriented reduction of rule-based network models IET Syst Biol 2008 2 5 342 351 http://dx.doi.org/10.1049/iet-syb:20070081 10.1049/iet-syb:20070081 19045829 PMC2628550

[B9] Saez-Rodriguez J Simeoni L Lindquist JA Hemenway R Bommhardt U Arndt B Haus UU Weismantel R Gilles ED Klamt S Schraven B A logical model provides insights into T cell receptor signaling PLoS Comput Biol 2007 3 8 e163 http://dx.doi.org/10.1371/journal.pcbi.0030163 10.1371/journal.pcbi.0030163 17722974 PMC1950951

[B10] Samaga R Saez-Rodriguez J Alexopoulos LG Sorger PK Klamt S The logic of EGFR/ErbB signaling: theoretical properties and analysis of high-throughput data PLoS Comput Biol 2009 5 8 e1000438 http://dx.doi.org/10.1371/journal.pcbi.1000438 10.1371/journal.pcbi.1000438 19662154 PMC2710522

[B11] Schlatter R Schmich K Vizcarra IA Scheurich P Sauter T Borner C Ederer M Merfort I Sawodny O ON/OFF and beyond–a boolean model of apoptosis PLoS Comput Biol 2009 5 12 e1000595 http://dx.doi.org/10.1371/journal.pcbi.1000595 10.1371/journal.pcbi.1000595 20011108 PMC2781112

[B12] Klamt S Saez-Rodriguez J Lindquist JA Simeoni L Gilles ED A methodology for the structural and functional analysis of signaling and regulatory networks BMC Bioinformatics 2006 7 56 http://dx.doi.org/10.1186/1471-2105-7-56 10.1186/1471-2105-7-56 16464248 PMC1458363

[B13] Klamt S Saez-Rodriguez J Gilles ED Structural and functional analysis of cellular networks with CellNetAnalyzer BMC Syst Biol 2007 1 2 http://dx.doi.org/10.1186/1752-0509-1-2 10.1186/1752-0509-1-2 17408509 PMC1847467

[B14] Samaga R von Kamp A Klamt S Computing combinatorial intervention strategies and failure modes in signaling networks J Comput Biol 2010 17 39 53 http://dx.doi.org/10.1089/cmb.2009.0121 10.1089/cmb.2009.0121 20078396

[B15] Saez-Rodriguez J Mirschel S Hemenway R Klamt S Gilles ED Ginkel M Visual setup of logical models of signaling and regulatory networks with ProMoT BMC Bioinformatics 2006 7 506 http://dx.doi.org/10.1186/1471-2105-7-506 10.1186/1471-2105-7-506 17109765 PMC1665465

[B16] Mirschel S Steinmetz K Rempel M Ginkel M Gilles ED PROMOT: modular modeling for systems biology Bioinformatics 2009 25 5 687 689 http://dx.doi.org/10.1093/bioinformatics/btp029 10.1093/bioinformatics/btp029 19147665 PMC2647835

[B17] Faeder JR Blinov ML Hlavacek WS Rule-based modeling of biochemical systems with BioNetGen Methods Mol Biol 2009 500 113 167 http://dx.doi.org/10.1007/978-1-59745-525-1_5 10.1007/978-1-59745-525-1_5 19399430

[B18] Blinov ML Faeder JR Goldstein B Hlavacek WS BioNetGen: software for rule-based modeling of signal transduction based on the interactions of molecular domains Bioinformatics 2004 20 17 3289 3291 http://dx.doi.org/10.1093/bioinformatics/bth378 10.1093/bioinformatics/bth378 15217809

[B19] Koschorreck M Gilles ED ALC: automated reduction of rule-based models BMC Syst Biol 2008 2 91 http://dx.doi.org/10.1186/1752-0509-2-91 10.1186/1752-0509-2-91 18973705 PMC2636783

[B20] Krivine J Danos V Benecke A Bouajjani A, Maler O Modelling Epigenetic Information Maintenance: a Kappa Tutorial Computer Aided Verification, Proceedings, Volume 5643 of Lecture Notes in Computer Science 2009 Springer-Verlag Berlin 17 32

[B21] Blinov M Yang J Faeder J Hlavacek W Priami C, Ingólfsdóttir A, Mishra B Graph Theory for Rule-Based Modeling of Biochemical Networks Transactions on Computational Systems Biology VII, Volume 4230 of Lecture Notes in Computer Science 2006 Riis Nielson H: Springer Berlin / Heidelberg 89 106 http://dx.doi.org/10.1007/11905455_5

[B22] Nag A Monine MI Blinov ML Goldstein B A detailed mathematical model predicts that serial engagement of IgE-Fc epsilon RI complexes can enhance Syk activation in mast cells J Immunol 2010 185 6 3268 3276 http://dx.doi.org/10.4049/jimmunol.1000326 10.4049/jimmunol.1000326 20733205 PMC3102320

[B23] Geier F Fengos G Iber D A computational analysis of the dynamic roles of talin, Dok1, and PIPKI for integrin activation PLoS One 2011 6 11 e24808 http://dx.doi.org/10.1371/journal.pone.0024808 10.1371/journal.pone.0024808 22110576 PMC3217926

[B24] Chylek LA Hu B Blinov ML Emonet T Faeder JR Goldstein B Gutenkunst RN Haugh JM Lipniacki T Posner RG Yang J Hlavacek WS Guidelines for visualizing and annotating rule-based models Mol Biosyst 2011 7 10 2779 2795 http://dx.doi.org/10.1039/c1mb05077j 10.1039/c1mb05077j 21647530 PMC3168731

[B25] Kocieniewski P Faeder JR Lipniacki T The interplay of double phosphorylation and scaffolding in MAPK pathways J Theor Biol 2012 295 116 124 http://dx.doi.org/10.1016/j.jtbi.2011.11.014 22123371 10.1016/j.jtbi.2011.11.014 PMC3417091

[B26] Yang J Monine MI Faeder JR Hlavacek WS Kinetic Monte Carlo method for rule-based modeling of biochemical networks Phys Rev E Stat Nonlin Soft Matter Phys 2008 78 3 Pt 1 031910 18851068 10.1103/PhysRevE.78.031910 PMC2652652

[B27] Colvin J Monine M Gutenkunst R Hlavacek W Von Hoff D Posner R RuleMonkey: software for stochastic simulation of rule-based models BMC Bioinformatics 2010 11 404 http://www.biomedcentral.com/1471-2105/11/404 10.1186/1471-2105-11-404 20673321 PMC2921409

[B28] Yang J Meng X Hlavacek WS Rule-based modeling and simulation of biochemical systems with molecular finite automata IET Syst Biol 2010 4 453 466 10.1049/iet-syb.2010.0015 21073243 PMC3070173

[B29] Kauffman SA Metabolic stability and epigenesis in randomly constructed genetic nets J Theor Biol 1969 22 3 437 467 10.1016/0022-5193(69)90015-0 5803332

[B30] Thomas R Boolean formalization of genetic control circuits J Theor Biol 1973 42 3 563 585 10.1016/0022-5193(73)90247-6 4588055

[B31] Thomas R D’Ari R Biological Feedback 1990 Boca Raton Florida: CRC Press

[B32] Mendoza L Thieffry D Alvarez-Buylla ER Genetic control of flower morphogenesis in Arabidopsis thaliana: a logical analysis Bioinformatics 1999 15 7-8 593 606 10487867 10.1093/bioinformatics/15.7.593

[B33] Albert R Othmer HG The topology of the regulatory interactions predicts the expression pattern of the segment polarity genes in Drosophila melanogaster J Theor Biol 2003 223 1 18 10.1016/S0022-5193(03)00035-3 12782112 PMC6388622

[B34] Christensen TS Oliveira AP Nielsen J Reconstruction and logical modeling of glucose repression signaling pathways in Saccharomyces cerevisiae BMC Syst Biol 2009 3 7 http://dx.doi.org/10.1186/1752-0509-3-7 10.1186/1752-0509-3-7 19144179 PMC2661888

[B35] Ryll A Samaga R Schaper F Alexopoulos LG Klamt S Large-scale network models of IL-1 and IL-6 signalling and their hepatocellular specification Mol Biosyst 2011 7 3253 3270 http://dx.doi.org/10.1039/c1mb05261f 10.1039/c1mb05261f 21968890

[B36] Cornish-Bowden A Fundamentals of Enzyme Kinetics 2004 London: Portland Press Ltd.

[B37] Karnaugh M The map method for synthesis of combinational logic circuits Transactions of the American Institute of Electrical Engineers 1953 72 9 593 599

[B38] Veitch EW A chart method for simplifying truth functions Proceedings of the 1952 ACM national meeting (Pittsburgh), 1952 New York, NY USA: ACM, ACM ’52 127 133 http://doi.acm.org/10.1145/609784.609801

[B39] McCluskey EJ Minimization of Boolean functions Bell Syst Tech J 1956 35 5 1417 1444

[B40] Ginkel M Kremling A Nutsch T Rehner R Gilles ED Modular modeling of cellular systems with ProMoT/Diva Bioinformatics 2003 19 9 1169 1176 http://bioinformatics.oxfordjournals.org/content/19/9/1169.abstract 10.1093/bioinformatics/btg128 12801880

[B41] Ward CW Lawrence MC Streltsov VA Adams TE McKern NM The insulin and EGF receptor structures: new insights into ligand-induced receptor activation Trends Biochem Sci 2007 32 3 129 137 http://dx.doi.org/10.1016/j.tibs.2007.01.001 10.1016/j.tibs.2007.01.001 17280834

[B42] Harris LA Hogg JS Faeder JR Compartmental rule-based modeling of biochemical systems. In Winter Simulation Conference, WSC ’09 Winter Simulation Conference 2009 908 919 http://dl.acm.org/citation.cfm?id=1995456.1995588

