# Supplementary material for: The Process-Interaction-Model: a common representation of rule-based and logical models allows studying signal transduction on different levels of detail
Source: BMC Bioinformatics. 2012 Sep 28;13:251. doi: 10.1186/1471-2105-13-251 (PMC3598730; doi:10.1186/1471-2105-13-251)
Supplement: Additional file 1 — Process-Interaction-Model of EGF insulin crosstalk (additional information). Additional file 1 lists the reaction rules and the associated parameters taken from [7] and used to elaborate the EGF insulin crosstalk example in the section “Results”. The process nodes of the PIM are correlated to the reaction rules in a tabular fashion. Furthermore, all parameter tables associated to the process nodes of the PIM are given. [file 1471-2105-13-251-S1.pdf]

reaction rule						P-Num
IR(0, 0, *, *)	+	Ins	↔	IR(I, 0, *, *)	k1, k-1	1
IR(0, 0, *, *)	+	Ins	↔	IR(0, I, *, *)	k1, k-1	2
IR(I, 0, *, *)	+	Ins	↔	IR(I, I, *, *)	k2, k-2	2
IR(0, I, *, *)	+	Ins	↔	IR(I, I, *, *)	k2, k-2	1
IR(0, 0, 0, *)			↔	IR(0, 0, P, *)	k3, k-3	3
IR(I, 0, 0, *)			↔	IR(I, 0, P, *)	k4, k-4	3
IR(0, I, 0, *)			↔	IR(0, I, P, *)	k4, k-4	3
IR(I, I, 0, *)			↔	IR(I, I, P, *)	k5, k-5	3
IR(*, *, P, *)	+	Shc(*)	↔	IR(*, *, Shc(*), *)	k6, k-6	5
IR(I, I, Shc(0), *)			↔	IR(I, I, Shc(P), *)	k7, k-7	6
IR(*, *, Shc(P), *)	+	Grb2(*)	↔	IR(*, *, Grb2(*), *)	k8, k-8	7
IR(*, *, Grb2(0), *)	+	SOS(0)	↔	IR(*, *, SOS(0), *)	k9, k-9	8
IR(*, *, Grb2(0), *)	+	SOS(P)	↔	IR(*, *, SOS(P), *)	k10, k-10	8
IR(*, *, SOS(0), *)			↔	IR(*, *, SOS(P), *)	k11, k-11	9
IR(0, 0, *, 0)			↔	IR(0, 0, *, P)	k12, k-12	4
IR(I, 0, *, 0)			↔	IR(I, 0, *, P)	k13, k-13	4
IR(0, I, *, 0)			↔	IR(0, I, *, P)	k13, k-13	4
IR(I, I, *, 0)			↔	IR(I, I, *, P)	k14, k-14	4
IR(*, *, *, P)	+	IRS(*)	↔	IR(*, *, *, IRS(*))	k15, k-15	10
IR(I, I, *, IRS(0))			↔	IR(I, I, *, IRS(P))	k16, k-16	11
IR(*, *, *, IRS(P))	+	Grb2(*)	↔	IR(*, *, *, Grb2(*))	k17, k-17	12
IR(*, *, *, Grb2(0))	+	SOS(0)	↔	IR(*, *, *, SOS(0))	k9, k-9	8
IR(*, *, *, Grb2(0))	+	SOS(P)	↔	IR(*, *, *, SOS(P))	k10, k-10	8
IR(*, *, *, SOS(0))			↔	IR(*, *, *, SOS(P))	k11, k-11	9
Shc(0)			↔	Shc(P)	k18, k-18	6
Shc(P)	+	Grb2(*)	↔	Shc(Grb2(*))	k8, k-8	7
Shc(Grb2(0))	+	SOS(0)	↔	Shc(SOS(0))	k9, k-9	8
Shc(Grb2(0))	+	SOS(P)	↔	Shc(SOS(P))	k10, k-10	8
Shc(SOS(0))			↔	Shc(SOS(P))	k11, k-11	9
Grb2(0)	+	SOS(0)	↔	Grb2(SOS(0))	k9, k-9	8
Grb2(0)	+	SOS(P)	↔	Grb2(SOS(P))	k10, k-10	8
Grb2(SOS(0))			↔	Grb2(SOS(P))	k11, k-11	9
SOS(0)			↔	SOS(P)	k19, k-19	9
IRS(0)			↔	IRS(P)	k20, k-20	11
IRS(P)	+	Grb2(*)	↔	IRS(Grb2(*))	k17, k-17	12
IRS(Grb2(0))	+	SOS(0)	↔	IRS(SOS(0))	k9, k-9	8
IRS(Grb2(0))	+	SOS(P)	↔	IRS(SOS(P))	k10, k-10	8
IRS(SOS(0))			↔	IRS(SOS(P))	k11, k-11	9
ER(0, *, *)	+	EGF	↔	ER(E, *, *)	k21, k-21	13
ER(*, 0, *)			↔	ER(*, P, *)	k22, k-22	14
ER(*, P, *)	+	Shc(*)	↔	ER(*, Shc(*), *)	k23, k-23	15
ER(*, Shc(0), *)			↔	ER(*, Shc(P), *)	k24, k-24	6
ER(*, Shc(P), *)	+	Grb2(*)	↔	ER(*, Grb2(*), *)	k8, k-8	7
ER(*, Grb2(0), *)	+	SOS(0)	↔	ER(*, SOS(0), *)	k9, k-9	8
ER(*, Grb2(0), *)	+	SOS(P)	↔	ER(*, SOS(P), *)	k10, k-10	8
ER(*, SOS(0), *)			↔	ER(*, SOS(P), *)	k11, k-11	9
ER(*, *, 0)			↔	ER(*, *, P)	k25, k-25	16
ER(*, *, P)	+	Grb2(*)	↔	ER(*, *, Grb2(*))	k26, k-26	17
ER(*, *, Grb2(0))	+	SOS(0)	↔	ER(*, *, SOS(0))	k9, k-9	8
ER(*, *, Grb2(0))	+	SOS(P)	↔	ER(*, *, SOS(P))	k10, k-10	8
ER(*, *, SOS(0))			↔	ER(*, *, SOS(P))	k11, k-11	9
ER(E, *, *)	+	ER(0, *, *)	↔	ER2(E, *, *, 0, *, *)	k27, k-27	18
ER(0, *, *)	+	ER(0, *, *)	↔	ER2(0, *, *, 0, *, *)	k28, k-28	18
ER(E, *, *)	+	ER(E, *, *)	↔	ER2(E, *, *, E, *, *)	k29, k-29	18
ER2(0, *, *, *, *, *)	+	EGF	↔	ER2(E, *, *, *, *, *)	k30, k-30	13
ER2(*, 0, *, *, *, *)			↔	ER2(*, P, *, *, *, *)	k31, k-31	14
ER2(*, Shc(0), *, *, *, *)			↔	ER2(*, Shc(P), *, *, *, *)	k32, k-32	6
ER2(*, P, *, *, *, *)	+	Shc(*)	↔	ER2(*, Shc(*), *, *, *, *)	k23, k-23	15
ER2(*, Shc(P), *, *, *, *)	+	Grb2(*)	↔	ER2(*, Grb2(*), *, *, *, *)	k8, k-8	7
ER2(*, Grb2(0), *, *, *, *)	+	SOS(0)	↔	ER2(*, SOS(0), *, *, *, *)	k9, k-9	8
ER2(*, Grb2(0), *, *, *, *)	+	SOS(P)	↔	ER2(*, SOS(P), *, *, *, *)	k10, k-10	8
ER2(*, SOS(0), *, *, *, *)			↔	ER2(*, SOS(P), *, *, *, *)	k11, k-11	9
ER2(*, *, 0, *, *, *)			↔	ER2(*, *, 0, *, *, *)	k33, k-33	16
ER2(*, *, P, *, *, *)	+	Grb2(*)	↔	ER2(*, *, Grb2(*), *, *, *)	k34, k-34	17
ER2(*, *, Grb2(0), *, *, *)	+	SOS(0)	↔	ER2(*, *, SOS(0), *, *, *)	k9, k-9	8
ER2(*, *, Grb2(0), *, *, *)	+	SOS(P)	↔	ER2(*, *, SOS(P), *, *, *)	k10, k-10	8
ER2(*, SOS(0), *, *, *, *)			↔	ER2(*, *, SOS(P), *, *, *)	k11, k-11	9

Figure 1: Rule-based model of EGF insulin crosstalk (adopted from [7]); last column corresponds process number in Process-Interaction-Model

k1	=	0.454545E0;
k2	=	0.909091E-2;
k3	=	0.9999E-4;
k4	=	0.125E1;
k5	=	(40/21);
k6	=	0.5E-1;
k7	=	0.47619E0;
k8	=	0.2E0;
k9	=	0.333333E-2;
k10	=	0.999001E-3;
k11	=	0.363636E0;
k12	=	0.1998E-2;
k13	=	(5/6);
k14	=	0.47619E0;
k15	=	0.4E-1;
k16	=	0.380952E0;
k17	=	0.25E0;
k18	=	0.990099E-4;
k19	=	0.4995E-4;
k20	=	0.990099E-4;
k21	=	0.727273E-1;
k22	=	0.39996E-4;
k23	=	(1/5);
k24	=	0.285714E0;
k25	=	0.19998E-4;
k26	=	0.6E-1;
k27	=	0.196262E-1;
k28	=	0.109989E-4;
k29	=	0.727273E0;
k30	=	(10/11);
k31	=	(10/11);
k32	=	0.380952E0;
k33	=	0.28125E0;
k34	=	0.133333E0;
k1d	=	0.454545E-1;
k2d	=	0.909091E-1;
k3d	=	0.9999E0;
k4d	=	0.25E0;
k5d	=	(2/21);
k6d	=	0.5E-1;
k7d	=	0.238095E-1;
k8d	=	1.E-1;
k9d	=	0.666667E-2;
k10d	=	0.999001E0;
k11d	=	0.363636E-1;
k12d	=	0.1998E1;
k13d	=	(1/6);
k14d	=	0.238095E-1;
k15d	=	0.4E-1;
k16d	=	0.190476E-1;
k17d	=	0.25E0;
k18d	=	0.990099E-2;
k19d	=	0.4995E-1;
k20d	=	0.990099E-2;
k21d	=	0.727273E0;
k22d	=	0.39996E0;
k23d	=	(4/5);
k24d	=	0.142857E-1;
k25d	=	0.19998E0;
k26d	=	0.3E-1;
k27d	=	0.280374E0;
k28d	=	0.109989E0;
k29d	=	0.727273E-1;
k30d	=	(1/11);
k31d	=	(1/11);
k32d	=	0.190476E-1;
k33d	=	0.1875E-1;
k34d	=	0.666667E-1;

Figure 2: Parameters for rule-based model of EGF and insulin crosstalk (adopted from [7])

Parameter table for *process 1* (binding)

Process 2	k_{fw}	k_{bw}	k_{eq}	y
0	k1	k-1	10	1
1	k2	k-2	0.1	1

Parameter table for *process 2* (binding)

Process 1	k_{fw}	k_{bw}	k_{eq}	y
0	k1	k-1	10	1
1	k2	k-2	0.1	1

Parameter table for *process 3* (modification)

Process 1	Process 2	k_{fw}	k_{bw}	k_{eq}	y
0	0	k3	k-3	0.0001	0
0	1	k4	k-4	5	1
1	0	k4	k-4	5	1
1	1	k5	k-5	20	1

Parameter table for *process 4* (modification)

Process 1	Process 2	k_{fw}	k_{bw}	k_{eq}	y
0	0	k12	k-12	0.001	0
0	1	k13	k-13	5	1
1	0	k13	k-13	5	1
1	1	k14	k-14	20	1

Parameter table for *process 5* (binding)

Process 3	k_{fw}	k_{bw}	k_{eq}	y
0	-	-	-	0
1	k6	k-6	1	1

Parameter table for *process 6* (modification)

Process 5	Process 15	k_{fw}	k_{bw}	k_{eq}	y
0	0	k18	k-18	0.01	0
0	1	k24	k-24	20	1
1	0	k7	k-7	20	1
1	1	-	-	-	1

Parameter table for *process 7* (binding)

Process 6	k_{fw}	k_{bw}	k_{eq}	y
0	-	-	-	0
1	k8	k-8	2	1

Parameter table for *process 8* (binding)

Process 9	k_{fw}	k_{bw}	k_{eq}	y
0	k9	k-9	0.5	1
1	k10	k-10	0.001	0

Parameter table for *process 9* (modification)

Process 8	k_{fw}	k_{bw}	k_{eq}	y
0	k19	k-19	0.001	0
1	k11	k-11	10	1

Parameter table for *process 10* (binding)

Process 4	k_{fw}	k_{bw}	k_{eq}	y
0	-	-	-	0
1	k15	k-15	1	1

Parameter table for *process 11* (modification)

Process 10	k_{fw}	k_{bw}	k_{eq}	y
0	k20	k-20	0.01	0
1	k16	k-16	20	1

Parameter table for *process 12* (binding)

Process 11	k_{fw}	k_{bw}	k_{eq}	y
0	-	-	-	0
1	k17	k-17	1	1

Parameter table for *process 13* (binding)

Process 18	k_{fw}	k_{bw}	k_{eq}	y
0	k21	k-21	0.1	1
1	k30	k-30	10	1

Parameter table for *process 14* (modification)

Process 18	k_{fw}	k_{bw}	k_{eq}	y
0	k22	k-22	0.0001	0
1	k31	k-31	10	1

Parameter table for *process 15* (binding)

Process 14	k_{fw}	k_{bw}	k_{eq}	y
0	-	-	-	0
1	k23	k-23	0.25	1

Parameter table for *process 16* (modification)

Process 18	k_{fw}	k_{bw}	k_{eq}	y
0	k25	k-25	0.0001	0
1	k33	k-33	15	1

Parameter table for *process 17* (binding)

Process 16	k_{fw}	k_{bw}	k_{eq}	y
0	-	-	-	0
1	k26	k-26	2	1

Parameter table for *process 18* (dimerization)

Process 13	Process 13'	k_{fw}	k_{bw}	k_{eq}	y
0	0	k28	k-28	0.0001	0
0	1	k27	k-27	0.07	-
1	0	-	-	-	-
1	1	k29	k-29	10	1
