# Supplementary figures and images for: The Process-Interaction-Model: a common representation of rule-based and logical models allows studying signal transduction on different levels of detail
Source: BMC Bioinformatics. 2012 Sep 28;13:251. doi: 10.1186/1471-2105-13-251 (PMC3598730; doi:10.1186/1471-2105-13-251)

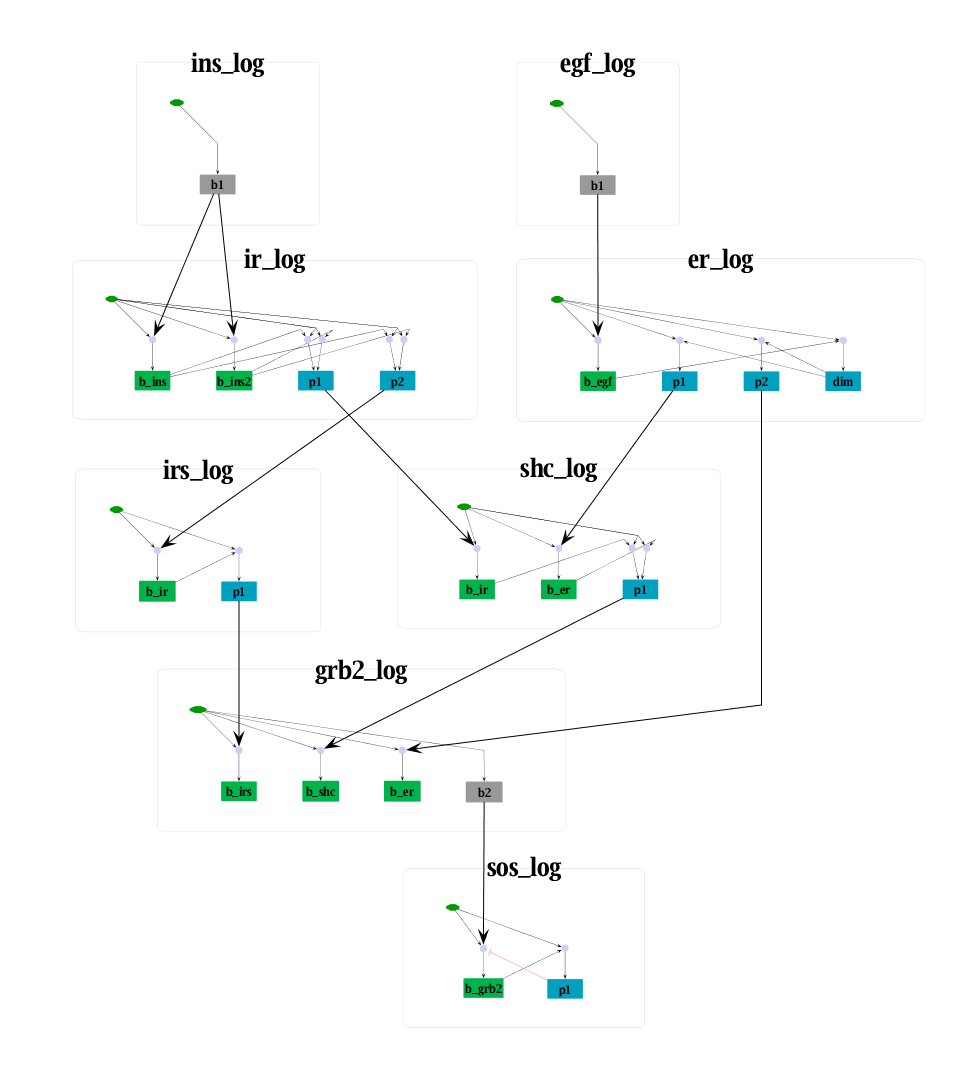

Supplement: Additional file 3 — Input files for the softwareCellNetAnalyzer (EGF insulin crosstalk). This archive contains all files of the logical model obtained from the PIM describing EGF insulin crosstalk (section Results) in CellNetAnalyzer format. CellNetAnalyzer is available from http://www.mpi-magdeburg.mpg.de/projects/cna/cna.html. [file 1471-2105-13-251-S3.zip › egf_insulin_crosstalk_map1.png]
